# Impact of GAP-43, Cx43 and actin expression on the outcome and overall survival in diffuse and anaplastic gliomas

**DOI:** 10.1038/s41598-023-29298-1

**Published:** 2023-02-04

**Authors:** Aleksandrs Krigers, Matthias Demetz, Patrizia Moser, Johannes Kerschbaumer, Konstantin R. Brawanski, Helga Fritsch, Claudius Thomé, Christian F. Freyschlag

**Affiliations:** 1 Department of Neurosurgery, Medical University of Innsbruck, Anichstrasse 35, 6020 Innsbruck, Austria; 2 Department of Neuropathology, University Hospital of Innsbruck, Tirol Kliniken, Austria; 3 Department of Anatomy, Histology and Embryology, Medical University of Innsbruck, Innsbruck, Austria

**Keywords:** CNS cancer, Tumour biomarkers, Glial biology

## Abstract

Distant intercellular communication in gliomas is based on the expansion of tumor microtubuli, where actin forms cytoskeleton and GAP-43 mediates the axonal conus growth. We aimed to investigate the impact of GAP-43 and actin expression on overall survival (OS) as well as crucial prognostic factors. FFPE tissue of adult patients with diffuse and anaplastic gliomas, who underwent first surgery in our center between 2010 and 2019, were selected. GAP-43, Cx43 and actin expression was analyzed using immunohistochemistry and semi-quantitatively ranked. 118 patients with a median age of 46 years (IqR: 35–57) were evaluated. 48 (41%) presented with a diffuse glioma and 70 (59%) revealed anaplasia. Tumors with higher expression of GAP-43 (*p* = 0.024, HR = 1.71/rank) and actin (*p* < 0.001, HR = 2.28/rank) showed significantly reduced OS. IDH1 wildtype glioma demonstrated significantly more expression of all proteins: GAP-43 (*p* = 0.009), Cx43 (*p* = 0.003) and actin (*p* < 0.001). The same was confirmed for anaplasia (GAP-43 *p* = 0.028, actin *p* = 0.029), higher proliferation rate (GAP-43 *p* = 0.016, actin *p* = 0.038), contrast-enhancement in MRI (GAP-43 *p* = 0.023, actin *p* = 0.037) and age (GAP-43 *p* = 0.004, actin *p* < 0.001; Cx43 n.s. in all groups). The intercellular distant communication network in diffuse and anaplastic gliomas formed by actin and GAP-43 is associated with a negative impact on overall survival and with unfavorable prognostic features. Cx43 did not show relevant impact on OS.

## Introduction

Gliomas, as the most common malignant primary brain tumors^1^, show a very heterogeneous course regarding progression and outcome^2^. Their behavior of diffuse and anaplastic gliomas World Health Organization (WHO) grade II and III, together known as lower-grade gliomas (LGG)^3,4^, depends on a wide range of radiologic, epidemiologic, and molecular factors. Despite an acknowledged negative impact of distinct genetic characteristics such as isocitrate dehydrogenase (IDH) status^2^, the effect of numerous pathological pathways, especially mechanisms to escape apoptosis induced by radiotherapy and to gain chemoresistance, remain poorly understood.

Intercellular communication has been shown as an important oncogenic element for growth and malignant development^5,6^. In gliomas, distant cell-to-cell interactions are possible due to microtubular networking^7–9^. This interaction is based on cytoskeletal cellular exvaginations—tumor microtubes (TMs), in which the main specific axonal conus component is neuronal growth associated protein 43 (GAP-43), also known as neuromodulin. In physiological conditions, GAP-43 is responsible for targeted migration and intercellular integration of neural precursors^10^.

Actin is a structural protein, that forms the cytoskeleton and thus influences a number of central cellular processes including cell adhesion, morphogenesis and mechano-transduction in various cancer types^11–13^. The TMs networking in gliomas are actin-rich as characteristic for most membrane nanotubes^7^. Thus, microtubular expansion is architecturally based on actin-cytoskeleton and its development is targeted by GAP-43 on the distal pole.

Connexin-43 (Cx43) allows physiological junctional cell-to-cell communication in astrocytes^14,15^ and neural precursors^16^. Normally, it is responsible for interchange of molecular signals and tissue homeostasis^15^. It was found on TMs^7^, however, its role remains mainly unclear^9,17^. It was described, that junctional intercellular activity might be an onco-promotive factor^5,18,19^.TMs allow effective brain colonization as well as regulate the electric and ligand exchange between remote parts of the tumor. In that way, even outlying glioma cells are interconnected to one large syncytium, resulting in a joint functional and mechanical network of tumor cells with potential by negative influence on resistance to treatment^7–9,20^. Nevertheless, an association between GAP-43, Cx43 and actin and survival has not been stated yet.

Thus, diffuse and anaplastic gliomas are heterogenous and correspondingly has a different behavior and outcome with numerous radiologic, epidemiologic, and molecular risk factors. We therefore aimed to investigate the expression of GAP-43, Cx43 and actin in relation these parameters as well as in relation to overall survival (OS) in diffuse and anaplastic gliomas.

## Materials and methods

All patients with histologically verified diffuse or anaplastic glioma, who underwent their first surgical treatment at our department between 2010 and 2019 were evaluated. These cases were recruited from an internal neuro-oncologic database and tissue bank, which have been approved by the institutional ethics committee (AN5220 329/4.4, informed consent was obtained preoperatively from every patient). Indication for surgery was based on international guidelines^21^, primarily favoring initial neurologically safe gross total resection. Further standardized treatment for each patient was discussed and announced in a multi-team neuro-oncological tumor board. Minimal age for inclusion was 18 years at the time of surgery.

After macroscopical inspection, the histopathological and molecular evaluation was performed according to clinical routine. The formalin-fixed paraffin-embedded (FFPE) tissue was processed and examined by experienced neuropathologists. The phenotype of glioma and sample conformity was checked in hematoxylin–eosin (HE) stain. WHO grading was performed according to phenotypical stratification as provided by the revised 4th WHO classification of the tumors of the central nervous system. If above 2 mitoses in 10 nuclei were detected in 10 consecutive high-powered fields, the tumor was signed as anaplastic according to the revised 4th WHO classification of the tumors of the central nervous system. With the usage of immunohistochemistry (IHC), the typical IDH1 R132H mutation was estimated. If this alteration was not confirmed, the deoxyribonucleic acid sequencing was done for patients under 40 years to verify wildtype IDH1. Proliferation index was quantitatively defined as the attitude of nuclei with positive MIB-1 expression in IHC in relation to all cells in that area. Nuclear ATP-dependent helicase (ATRX), epidermal growth factor receptor (EGFR) and p53 were tested by IHC as well.

GAP-43, Cx43 and actin expression were revealed with IHC, which was automatically performed with the usage of specific antibodies (Abcam EP890Y anti-GAP43 1:1000, Abcam P17302 anti-Connexin-43 1:2000 and Abcam EPR16769 anti-Actin 1:4000). Positive and negative IHC controls were performed. The expression of actin and GAP-43 was semi-quantitatively defined in glioma cellular fractions with “0” for lack of any expression, “1” for light expression, “2” for intermediate expression, e.g., more than light and less than strong, and “3” for strong expression that is equal to cortex: examples are provided in supplement 1. Additional HE stained slides were performed consecutively to verify the glioma position in the sample and tissue representability. The examination was performed by 2 specialists and in case of inconsonant evaluation, the matched result was approved after bilateral discussion.

The epidemiological, clinical and follow-up data were acquired from the prospective neuro-oncological database. Magnetic resonance imaging (MRI) before surgery was performed as a standard procedure in our institution. The positive volumetric measurement of the contrast enhancement (CE) in T1-sequence, as well as native in T2, Fluid-attenuated inversion recovery (FLAIR) and Diffusion-weighted imaging (DWI) weighted sequences was manually performed with ITK-SNAP software (v.3.8.0 for Mac OS, UPenn and UNC dev.).

Descriptive and analytic statistic evaluation was performed with IBM SPSS Statistics (IBM SPSS Statistics for Mac OS, Version 27.0. Armonk, NY: IBM Corp.). Not parametric scale and ranked data were presented as median with corresponding interquartile range (IqR). Monovariate analysis was done with Spearman correlation test, Mann–Whitney U-test and Chi^2^-test. Overall survival hazards were evaluated with Cox regression and illustrated by Kaplan–Meier graphs. The confidence interval and significance level were set as 95%.

### Ethics approval

The study was performed in accordance with the ethical standards as laid down in the 1964 Declaration of Helsinki and its later amendments or comparable ethical standards. The database and tissue bank are approved by the ethics committee of Medical University of Innsbruck (AN5220 329/4.4).

### Consent to participate

Written informed content was acquired from the participants.

## Results

### Descriptive evaluation

A total of 161 patients were found eligible for the initial investigation. 43 patients had to be excluded due to insufficient amount of FFPE tissue available, so that IHC could not be performed. Finally, the evaluation was performed for 118 patients. The detailed stratification of these cases according to the revised 4th WHO classification of the tumors of central nervous system are provided in Table 1.

**Table 1:** Stratification of diffuse and anaplastic gliomas in our cohort according to the revised 4th WHO classification of the tumors of central nervous system from 2016.

Tumor type	n
Anaplastic Astrocytoma, IDH wildtype	32
Anaplastic Astrocytoma, IDH mutant	18
Diffuse Astrocytoma, IDH mutant	12
Oligodendroglioma, IDH mutant and 1p/19q-codeleted	10
Anaplastic Oligoastrocytoma, NOS	9
Anaplastic Oligodendroglioma, IDH mutant and 1p/19q-codeleted	9
Oligodendroglioma, NOS	9
Anaplastic Oligodendroglioma, NOS	8
Diffuse Astrocytoma, IDH wildtype	8
Oligoastrocytoma, NOS	3

The gender distribution favored men with 70 cases (59%) vs. women with 48 cases (41%), median age was 46 years (IqR: 35–57, absolute range: 19–86). 108 patients (92%) underwent surgical resection and 10 patients (8%) navigated biopsy. Median postoperative follow-up amounted to 32 months (IqR: 17–61).

The vast majority of tumors (96%) was limited to one hemisphere (right: n = 52; left: n = 61), whereas only 5 tumors (4%) occurred bilaterally. 66 (56%) gliomas were located in the frontal lobe, 23 (20%) parietally, 49 (42%) temporally and 6 (5%) occipitally. Insular localization was found in 26 (22%) cases, thalamus or basal ganglia in 20 (17%), brain stem in 4 (3%) and cerebellum in 5 (4%). 17 (14%) gliomas invaded the corpus callosum.

**Figure 1:**
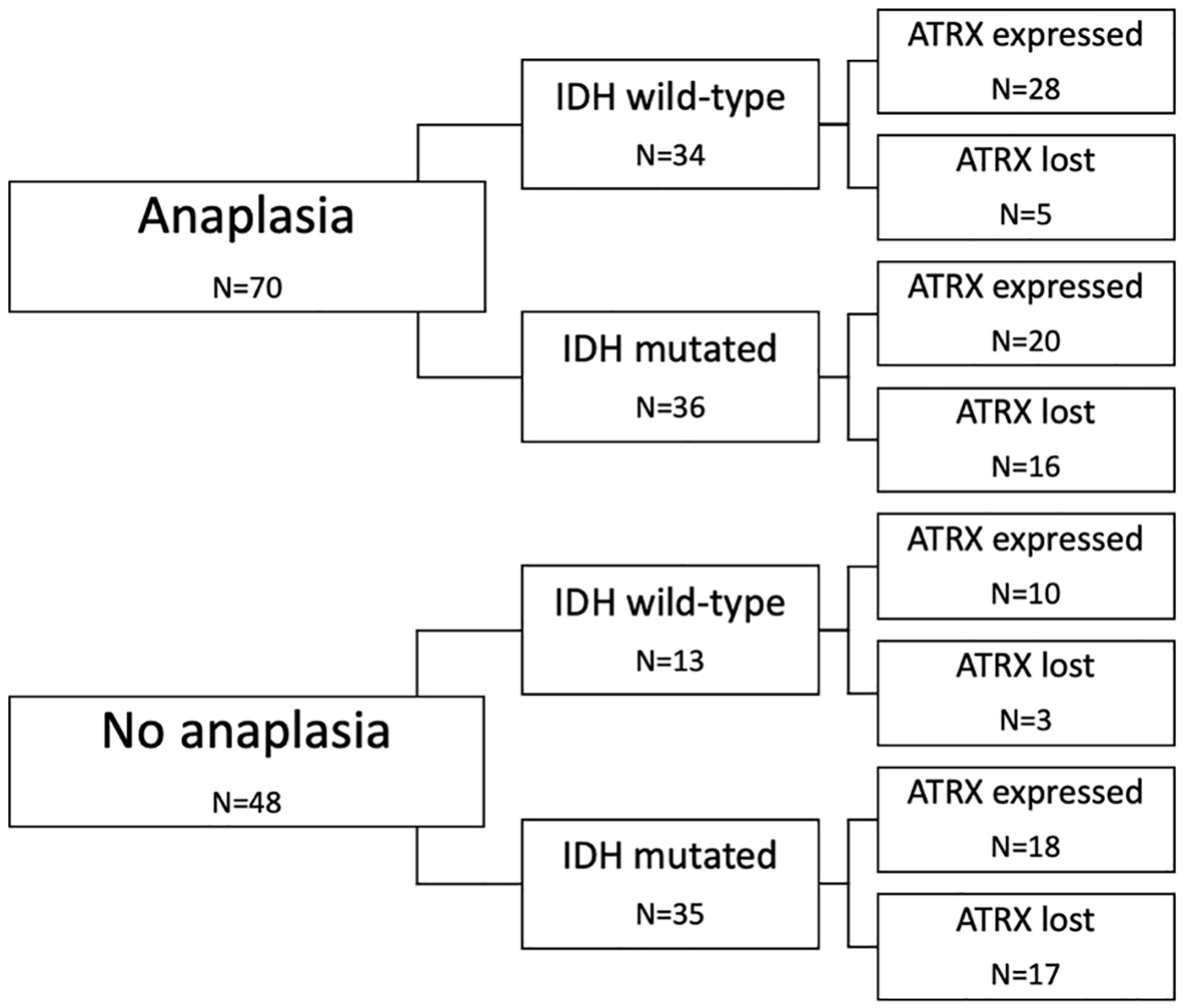
The stratification of anaplasia, IDH and nuclear ATRX status of our cohort.

Regarding the malignancy of LGG, 48 (41%) tumors were diffuse and 70 (59%) anaplastic, whereas 20 of them showed focal anaplasia and others were entirely anaplastic. Median expression of MIB-1 proliferation marker amounted to 14% (IqR: 6–24). IDH1 status could be determined in all patients and was mutated in 71 (60%) and wildtype in 47 (40%) cases. EGFR status was identified in all patients as well with no expression in 36 (31%) and confirmed overexpression in 82 (69%) cases. Nuclear ATRX production was lost in 41 (35%) and expressed in 76 (64%) LGGs, in one case the result was not plausible. The detailed distribution of the neuropathological features is shown in Fig. 1.

**Figure 2:**
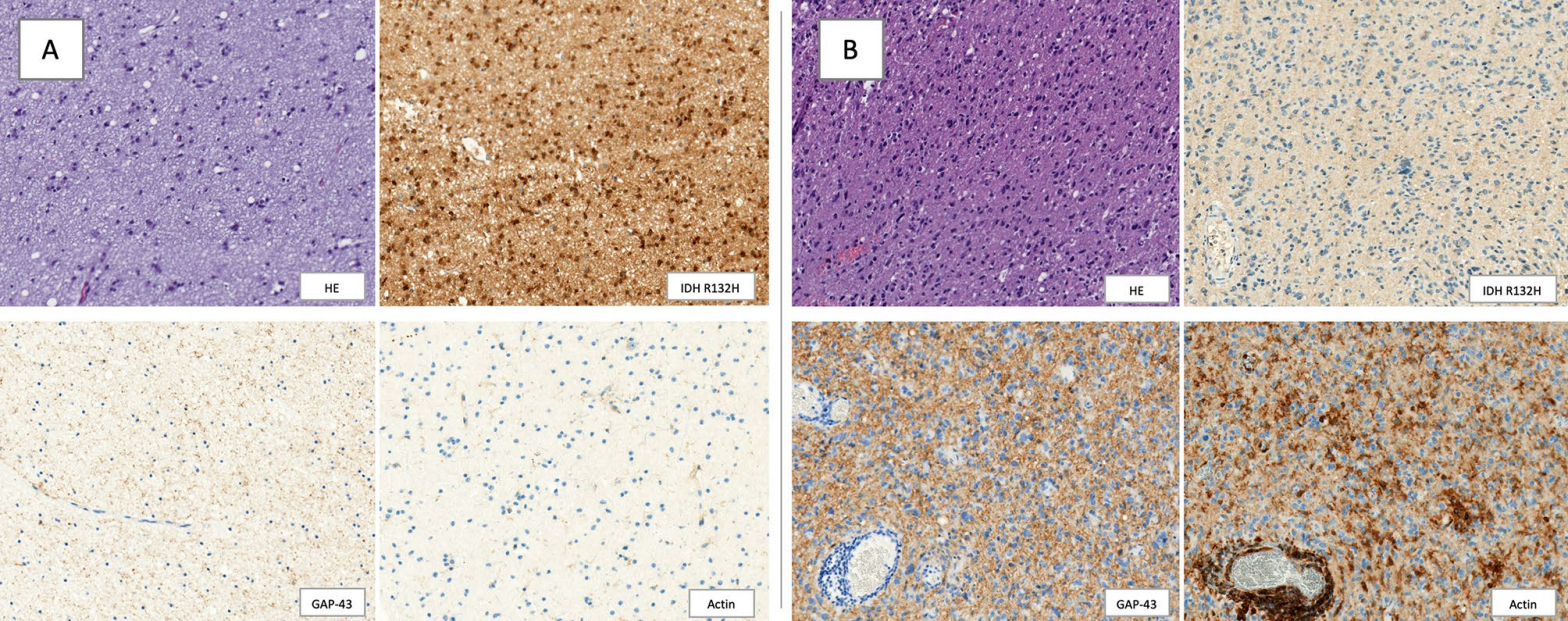
Glioma samples: (**A**) with mutated IDH shows light GAP-43 and actin expression; (**B**) with wildtype IDH shows strong GAP-43 and actin expression. HE slide is followed by IHC for IDH1 R132H mutation, GAP-43 and actin expression (Precipoint M8, 400×).

The expression stratification of GAP-43, Cx43 and actin as fundamental components of TMs is shown in Table 2. Greater number of tumors showed higher expression of GAP-43 and Cx43, as well as lower expression of actin. Examples of the expression of GAP-43 and actin communication proteins for IDH1 mutated and wildtype sample are provided in Fig. 2.

**Table 2:** Semi-quantitative stratification of GAP-43, Cx43 and actin expression.

		GAP-43, N (%)	Cx43, N (%)	Actin, N (%)
0	No expression	5 (4)	6 (5)	50 (43)
1	Slight expression	16 (14)	19 (16)	28 (24)
2	Intermediate expression	42 (36)	35 (29)	22 (19)
3	Strong expression	54 (46)	58 (50)	17 (14)

### Risk factor analysis

The significant results of the monovariate analysis are shown in Table 3. No gender-specific differences were identified. Age, however, directly correlated with the expression of GAP-43 (r = 0.262) and actin (r = 0.324).

**Table 3:** The association of GAP-43, Cx43 and actin expression with epidemiologic, neuropathological and radiological variables.

	GAP-43, p^†^	Cx43, p^†^	Actin, p†
Gender	n.s	n.s	n.s
Age	0.004*	n.s	< 0.001*
Anaplasia	0.028*	n.s	0.029*
IDH1 wild-type	0.009*	0.003*	< 0.001*
ATRX nuclear expression	0.037*	n.s	n.s
EGFR decreased expression	n.s	n.s	0.031*
MIB-1	0.016*	n.s	0.038*
T1 CE volume	n.s	0.008**	< 0.001*
T2 volume	n.s	n.s	n.s
FLAIR volume	n.s	n.s	n.s
DWI volume	n.s	n.s	n.s
Parietal localization	n.s	n.s	0.002*

*Direct association,

**Opposite association.

^†^*p* < 0.05 is considered as significant, otherwise “n.s.” is provided.

In case of anaplasia or wildtype IDH, expression of all three focus proteins was upregulated. Lost ATRX nuclear expression was related to lower GAP-43 and no expression of EGFR to lower actin expression. The proliferation index directly correlated with the expression of both markers: GAP-43 (r = 0.241) and actin (r = 0.208).

A higher T1 CE tumor volume on preoperative MRI directly correlated with the expression of actin (r = 0.336) and opposite to Cx43 (− 0.242), whereas no correlations to native volumes were identified. Considering tumor position or side, only parietal localization was related to a higher actin expression.

While expression of GAP-43 was significantly lower in incidental LGG than in patients with symptomatic LGG (*p* = 0.001), no associations was found for actin or Cx43 (*p*–n.s.).

### Assessment of OS

The Cox regression models for overall survival considering expression of three focus proteins were established: increased expression of GAP-43 and actin showed a significant impact for worse outcome. GAP-43 presented with a hazard ratio (HR) of 1.712 (CI95% 1.072–2.732, *p* = 0.024) and actin with a HR of 2.276 (CI95% 1.663–3.115, *p* < 0.001) per semi-quantitative step of expression.

**Figure 3:**
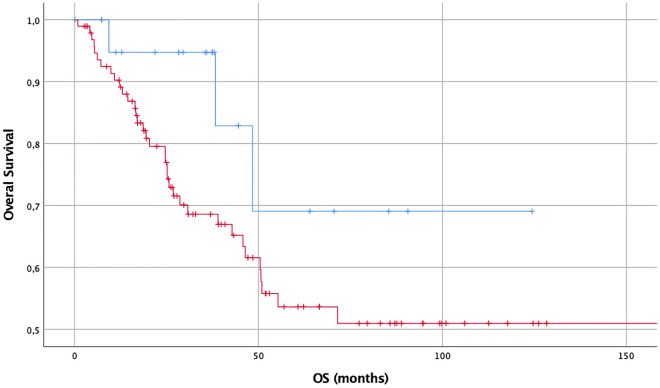
OS (months) in diffuse and anaplastic gliomas considering expression of GAP-43: no or slight expression (blue, n = 21) versus intermediate or strong expression (red, n = 96), demonstrating worse outcome for tumors, which are expressing more GAP-43.

**Figure 4:**
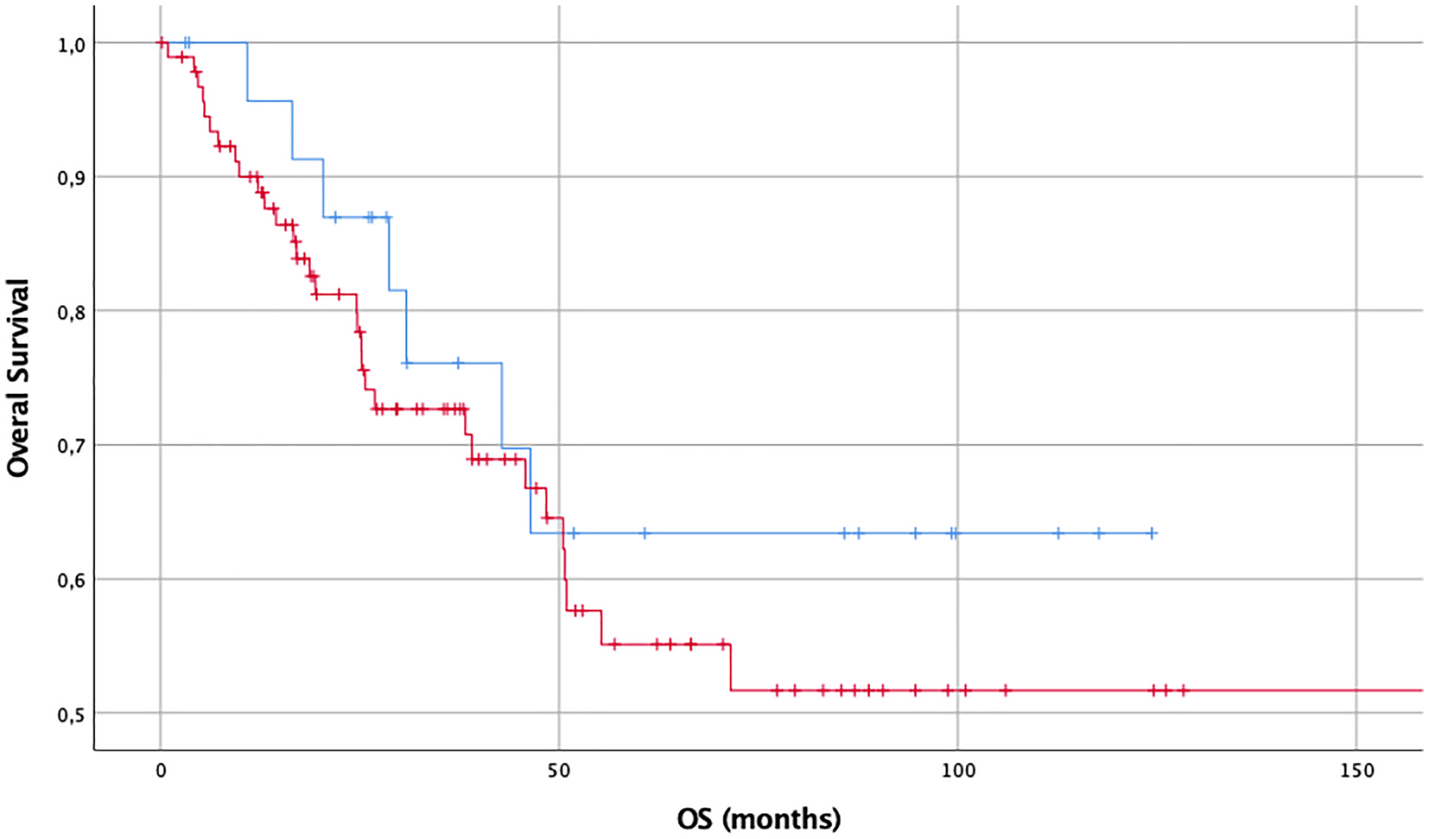
OS (months) in diffuse and anaplastic gliomas considering expression of Cx43: no or slight expression (blue, n = 25) versus intermediate or strong expression (red, n = 93), demonstrating no significant survival differences between these tho groups.

**Figure 5:**
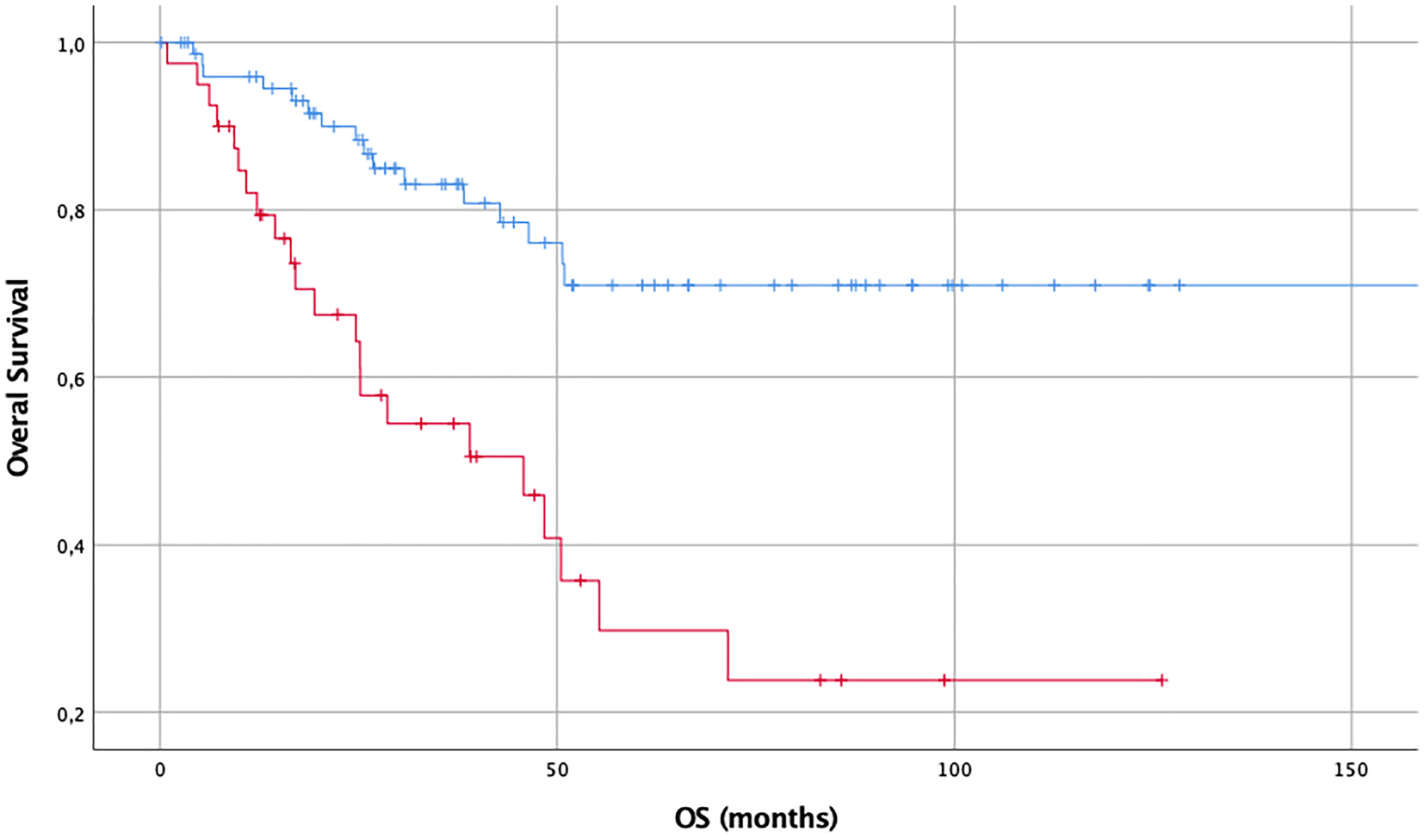
OS (months) in diffuse and anaplastic gliomas considering expression of actin: no or slight expression (blue, n = 78) vs. intermediate or strong expression (red, n = 39), demonstrating worse survival for tumors with higher actin expression.

The concordant results are presented in the Kaplan–Meier curves below—Figs. 3, 4 and 5.

## Discussion

In this study, we analyzed the expression of GAP-43, Cx43 and actin as fundamental markers of TMs-mediated distant intercellular communication in samples of 118 patients, harboring diffuse or anaplastic glioma. We demonstrated that increased expression of GAP-43 and actin proteins was associated with a significantly worse outcome and shorter OS. Moreover, prognostically unfavorable factors like greater age, CE volume in MRI, elevated proliferation index, anaplasia and wildtype IDH status were related to the higher expression of both GAP-43 and actin.

Microtubular-based networking in glioma provide tumor migration and physiological unification; thus, stimulating its expansion and resistance against specific treatments^7–9,19^. GAP-43 on the axonal conus leads the growth and actin forms the TMs cytoskeleton. Data concerning clinical implications of these key communication proteins, however, are limited. Previous research was mainly focused on basic in vitro studies using glioblastoma cell lines, which allow only limited translation to clinical neuro-oncology^5,7–10,13,22^.

The baseline epidemiologic and radiologic characteristics of gliomas in our cohort, such as median age, gender balance and tumor location, are consistent with the existing literature^1,23,24^ and, hence, is representative. The LGG in this study also presented with the stratification of neuropathological features, especially IDH, ATRX and EGFR, concordant to previously published data^25–27^.

More than 80% of diffuse and anaplastic gliomas showed moderate or strong GAP-43 expression. GAP-43 was found to be physiologically upregulated in axonal growth cones during neurite extension^28–30^. Knockdown of GAP-43 resulted in to impaired neural progenitor cell migration^8,31^. In analogy, glioma cells might achieve TMs outgrowth and taxis. This proposed role of GAP-43 in malignant processes leading to progression in LGG was demonstrated in our study: increased expression of GAP-43 was associated with significantly shorter OS. Thus, in case of strong expression of GAP-43, the hazards to decease were more than 2.1 times higher compared to no expression at all.

At the same time, actin was habitually low-expressed in the samples of our patients: in 2/3 cases either no or only light expression was detected. Actin is a structural protein, that mechanically forms the cytoskeleton, including the internal architectural construct of TMs. This finding could thus be interpreted that the TMs were just being developed. Hence, the axonal cones of TMs afforded by GAP-43 are already growing, but TMs still stay immature, i.e., not elongated and incompletely mechanically formed. Interestingly, in case of strong actin expression, the OS sank dramatically: the hazards to decease got 3.8 times higher compared to no expression.

The suspected negative impact of GAP-43- and actin-mediated TMs also matched our further results, as the expression of both focus proteins was associated with known prognostically unfavorable factors like anaplasia^32^, wildtype IDH^33^, higher age^34^, elevated replication rate^35^ or CE volume in MRI for actin^36–38^. Overexpressed EGFR is considered to be a negative prognostic factor in case of gliomas^39,40^, which related to higher actin expression in our cohort. In these cases, the intercellular distant networking due to advanced TMs development may have been more advanced.

According to descriptive assessment, greater number of tumors showed higher expression of GAP-43 and lower expression of actin. In analytical assessment, both proteins were associated with negative prognostic features. Whereas, hazards and risks were higher for actin, which is concordant to descriptive assessment. It could be interpreted as higher actin expression is not so frequent, but if it is the case then this glioma is associated with remarkably higher hazards and risks.

We showed a difference in the expression of GAP-43 between symptomatic and incidental gliomas. The less developed GAP-43 expression in asymptomatic cases is concordant to the more favorable outcome of these patients^41–43^. It confirms that these tumors were just detected earlier, e.g., they did not develop the high-level intercellular communication yet.

Interestingly, significantly higher expression of actin was present in parietal LGG. Although this finding remains unexplained, it has been previously shown that certain glioma subgroups occur more often in specific brain regions, perhaps due to different precursor cells or local tissue arrangements^44,45^.

On the other hand, Cx43 expression showed no significant impact on OS and relevant associations with other, radiological or neuropathological factors. Only direct association with IDH wildtype status was reveled, which is prognostically unfavorable factor.

Our study has limitations. This is a single-center study and the population used here may not be representative for other cohorts. However, the stratification of key glioma features was concordant to epidemiology and previous studies. The WHO classification of glioma was performed according to revised 4th classification. Still, canonic role of the IDH status remained unchanged even after the classification update. According to updated 5th WHO classification of CNS tumors, some wildtype gliomas (with TERT promoter mutation or EGFR gene amplification or + 7/− 10 chromosome copy number changes), that were classified as °II and °III before are now considered as “molecular” glioblastoma. As we used revised 4th WHO classification of CNS tumors, we are not able to distinguish these cases. IHC allows only semi-quantitative evaluation. Nevertheless, the test and assessment protocol were standardized, thus, the comparison of tumors inside the cohort stayed reliable.

A well-structured and developed distant intercellular networking using TMs seems to promote an aggressive behavior of diffuse and anaplastic gliomas. By facilitating cell invasion and exchange of resistance to treatment, this could result in a worse prognosis of patients harboring LGG. Further studies are necessary to better understand the underlying cellular mechanisms and pathways as well as to identify potential therapeutic approaches.

## Conclusion

In this study, a significantly worse outcome with a shortened OS of LGG patients was demonstrated in case of increased GAP-43 and actin expression. Moreover, prognostically unfavorable factors like anaplasia, high proliferation index, wildtype IDH, CE in MRI and age was associated with the higher expression of these communication proteins. Cx43 did not show relevant impact on OS. Our findings suggest that advanced distant intercellular communication via TMs could be a relevant factor in oncological progression with consequently worse outcome.

## Supplementary Information

Supplementary Information.

## Data Availability

The raw data was generated in authors’ institution. The data that support the findings of this study are available on reasonable request from the corresponding author. The data are not publicly available due their containing information that could compromise the privacy of research participants.

## References

[1] Ostrom QT (2020). CBTRUS statistical report: Primary brain and other central nervous system tumors diagnosed in the United States in 2013–2017. Neuro Oncol..

[2] Louis DN (2021). The 2021 WHO Classification of tumors of the central nervous system: A summary. Neuro Oncol..

[3] Cancer Genome Atlas Research Network (2015). Comprehensive, integrative genomic analysis of diffuse lower-grade gliomas. N. Engl. J. Med..

[4] Gittleman H, Sloan AE, Barnholtz-Sloan JS (2020). An independently validated survival nomogram for lower-grade glioma. Neuro Oncol..

[5] Sinyuk M, Mulkearns-Hubert EE, Reizes O, Lathia J (2018). Cancer connectors: Connexins, gap junctions, and communication. Front. Oncol..

[6] Brucher BL, Jamall IS (2014). Cell-cell communication in the tumor microenvironment, carcinogenesis, and anticancer treatment. Cell Physiol. Biochem..

[7] Osswald M (2015). Brain tumour cells interconnect to a functional and resistant network. Nature.

[8] Osswald M, Solecki G, Wick W, Winkler F (2016). A malignant cellular network in gliomas: Potential clinical implications. Neuro Oncol..

[9] Weil S (2017). Tumor microtubes convey resistance to surgical lesions and chemotherapy in gliomas. Neuro Oncol..

[10] Haag D (2012). Nos2 inactivation promotes the development of medulloblastoma in Ptch1(+/-) mice by deregulation of Gap43-dependent granule cell precursor migration. PLoS Genet.

[11] Shishkin S, Eremina L, Pashintseva N, Kovalev L, Kovaleva M (2016). Cofilin-1 and other ADF/Cofilin superfamily members in human malignant cells. Int. J. Mol. Sci..

[12] Kessler J (2019). Radiosensitization and a less aggressive phenotype of human malignant glioma cells expressing isocitrate dehydrogenase 1 (IDH1) mutant protein: Dissecting the mechanisms. Cancers (Basel).

[13] Tojkander S, Gateva G, Lappalainen P (2012). Actin stress fibers–assembly, dynamics and biological roles. J. Cell Sci..

[14] Giaume C (1991). Gap junctions in cultured astrocytes: Single-channel currents and characterization of channel-forming protein. Neuron.

[15] Zhao Y, Xin Y, He Z, Hu W (2018). Function of connexins in the interaction between glial and vascular cells in the central nervous system and related neurological diseases. Neural Plast.

[16] Marins M (2009). Gap junctions are involved in cell migration in the early postnatal subventricular zone. Dev. Neurobiol..

[17] Sin WC, Crespin S, Mesnil M (2012). Opposing roles of connexin43 in glioma progression. Biochim. Biophys. Acta.

[18] Aasen T (2019). Connexins in cancer: Bridging the gap to the clinic. Oncogene.

[19] Krigers A (2021). The advanced development of Cx43 and GAP-43 mediated intercellular networking in IDH1 wildtype diffuse and anaplastic gliomas with lower mitotic rate. J. Cancer Res. Clin. Oncol..

[20] Sontheimer H (2015). Brain cancer: Tumour cells on neighbourhood watch. Nature.

[21] Weller M (2021). EANO guidelines on the diagnosis and treatment of diffuse gliomas of adulthood. Nat. Rev. Clin. Oncol..

[22] Sinyuk, M. & Lathia, J. D. in *Intercellular Communication in Cancer* (ed Mustapha Kandouz) Ch. Chapter 2, 29–41 (Springer Netherlands, 2015).

[23] Skjulsvik AJ (2020). Is the anatomical distribution of low-grade gliomas linked to regions of gliogenesis?. J. Neurooncol..

[24] Hess KR, Broglio KR, Bondy ML (2004). Adult glioma incidence trends in the United States, 1977–2000. Cancer.

[25] Eckel-Passow JE (2015). Glioma groups based on 1p/19q, IDH, and TERT promoter mutations in tumors. N. Engl. J. Med..

[26] Turkalp Z, Karamchandani J, Das S (2014). IDH mutation in glioma: New insights and promises for the future. JAMA Neurol..

[27] Fukuma R (2019). Prediction of IDH and TERT promoter mutations in low-grade glioma from magnetic resonance images using a convolutional neural network. Sci. Rep..

[28] Korshunova I (2007). GAP-43 regulates NCAM-180-mediated neurite outgrowth. J. Neurochem..

[29] Shen Y, Meiri K (2013). GAP-43 dependency defines distinct effects of netrin-1 on cortical and spinal neurite outgrowth and directional guidance. Int. J. Dev. Neurosci..

[30] Strittmatter SM, Igarashi M, Fishman MC (1994). GAP-43 amino terminal peptides modulate growth cone morphology and neurite outgrowth. J. Neurosci..

[31] Jung E (2019). Emerging intersections between neuroscience and glioma biology. Nat. Neurosci..

[32] Pujadas E, Chen L, Rodriguez FJ (2019). Pathologic and molecular aspects of anaplasia in circumscribed gliomas and glioneuronal tumors. Brain Tumor Pathol..

[33] Houillier C (2010). IDH1 or IDH2 mutations predict longer survival and response to temozolomide in low-grade gliomas. Neurology.

[34] Krigers A, Demetz M, Thome C, Freyschlag CF (2021). Age is associated with unfavorable neuropathological and radiological features and poor outcome in patients with WHO grade 2 and 3 gliomas. Sci. Rep..

[35] Johannessen AL, Torp SH (2006). The clinical value of Ki-67/MIB-1 labeling index in human astrocytomas. Pathol. Oncol. Res..

[36] Hempel JM (2018). Contrast enhancement predicting survival in integrated molecular subtypes of diffuse glioma: An observational cohort study. J. Neurooncol..

[37] Kanamori M (2016). Malignant transformation of diffuse astrocytoma to glioblastoma associated with newly developed BRAF V600E mutation. Brain Tumor Pathol..

[38] Pallud J (2009). Prognostic significance of imaging contrast enhancement for WHO grade II gliomas. Neuro Oncol..

[39] Li J, Liang R, Song C, Xiang Y, Liu Y (2018). Prognostic significance of epidermal growth factor receptor expression in glioma patients. Onco Targets Ther..

[40] Saadeh FS, Mahfouz R, Assi HI (2018). EGFR as a clinical marker in glioblastomas and other gliomas. Int. J. Biol. Markers.

[41] Ius T (2020). Incidental low-grade gliomas: Single-institution management based on clinical, surgical, and molecular data. Neurosurgery.

[42] Ius T (2022). The benefit of early surgery on overall survival in incidental low-grade glioma patients: a multicenter study. Neuro Oncol..

[43] Zeng L (2021). A survival analysis of surgically treated incidental low-grade glioma patients. Sci. Rep..

[44] Zlatescu MC (2001). Tumor location and growth pattern correlate with genetic signature in oligodendroglial neoplasms. Cancer Res..

[45] Duffau H, Capelle L (2004). Preferential brain locations of low-grade gliomas. Cancer.

