# Supplementary material for: Impact of GAP-43, Cx43 and actin expression on the outcome and overall survival in diffuse and anaplastic gliomas
Source: Sci Rep. 2023 Feb 4;13:2024. doi: 10.1038/s41598-023-29298-1 (PMC9899260; doi:10.1038/s41598-023-29298-1)

**Supplement 1.** Examples of semi-qualitative evaluation of GAP-43, Cx43 and actin expression in diffuse and anaplastic gliomas: “0” stands for no expression, “1” for light expression, “2” for intermediate expression and “3” for strong expression.


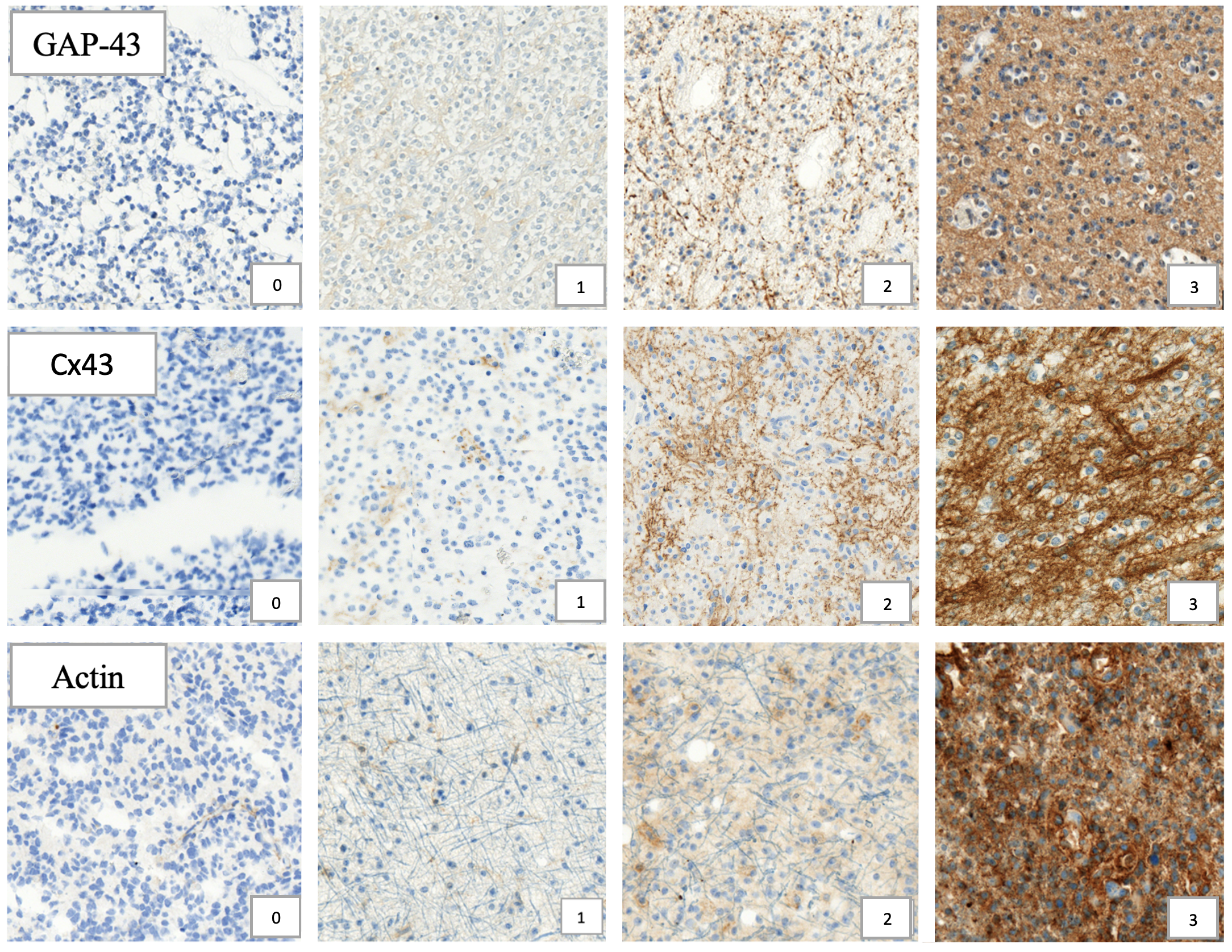

Supplement: Supplementary file 1 — Supplementary Information. [file 41598_2023_29298_MOESM1_ESM.docx]
